# Supplementary material for: Cognitive Effects of Toxoplasma and CMV Infections: A Cross-Sectional Study of 557 Young Adults Considering Modulation by Sex and Rh Factor
Source: Pathogens. 2024 Apr 28;13(5):363. doi: 10.3390/pathogens13050363 (PMC11124290; doi:10.3390/pathogens13050363)
Supplement: Supplementary file 1 [file pathogens-13-00363-s001.zip › pathogens-2920893-supplementary.pdf]

**Supplementary table**

Table S1 Relations between sex, toxoplasmosis, CMV infection, and age and variables related to cognitive performance – the effect size (Cohen d) and significance of t-test (p)

| | Sex | | *Toxoplasma* | | CMV | |
|---|---|---|---|---|---|---|
| | p | Cohen d | p | Cohen d | p | Cohen d |
| **I-S-T 2000 R (intelligence and knowledge: N = 533, memory: N = 305)** | | | | | | |
| General intelligence | 0.001 | 0.307 | 0.053 | -0.213 | 0.744 | 0.029 |
| General knowledge | 0.000 | 0.731 | 0.636 | -0.051 | 0.760 | -0.027 |
| Verbal intelligence | 0.043 | 0.187 | 0.640 | -0.055 | 0.045 | 0.178 |
| Verbal knowledge | 0.000 | 0.584 | 0.640 | 0.052 | 0.151 | 0.127 |
| Numerical intelligence | 0.000 | 0.430 | 0.091 | -0.195 | 0.792 | -0.023 |
| Numerical knowledge | 0.000 | 0.441 | 0.087 | -0.186 | 0.206 | -0.112 |
| Figural intelligence | 0.577 | 0.051 | 0.096 | -0.184 | 0.794 | -0.023 |
| Figural knowledge | 0.000 | 0.692 | 0.729 | 0.037 | 0.667 | -0.038 |
| Crystallized intelligence | 0.000 | 0.725 | 0.954 | 0.006 | 0.960 | 0.004 |
| Fluid intelligence | 0.017 | 0.220 | 0.070 | -0.208 | 0.882 | 0.013 |
| Associative memory | 0.000 | -0.426 | 0.841 | -0.025 | 0.121 | -0.181 |
| **Meili memory tests (location: N = 457, free recall: N = 454, yes-no: N = 456)** | | | | | | |
| Spatial memory | 0.000 | -0.426 | 0.339 | -0.121 | 0.198 | -0.125 |
| Free recall memory | 0.688 | -0.038 | 0.037 | 0.203 | 0.840 | -0.020 |
| Recognition memory | 0.048 | -0.194 | 0.315 | -0.131 | 0.284 | -0.104 |
| **Simple reaction time test (N = 466)** | | | | | | |
| Reaction time | 0.018 | -0.230 | 0.399 | 0.118 | 0.200 | 0.123 |
| **Prepulse tests (acoustic: N = 415, visual: 420)** | | | | | | |
| Acoustic test, prepulse | 0.555 | -0.061 | 0.145 | 0.186 | 0.611 | 0.051 |
| Acoustic test, no prepulse | 0.728 | -0.034 | 0.259 | 0.136 | 0.969 | -0.004 |
| Visual test, prepulse | 0.443 | -0.078 | 0.995 | -0.001 | 0.021 | 0.230 |
| Visual test, no prepulse | 0.122 | -0.160 | 0.821 | 0.031 | 0.060 | 0.188 |
| **Stroop tests (test A: N = 419, test B: N = 407)** | | | | | | |
| Test A, correct no-reactions | 0.391 | -0.092 | 0.381 | 0.114 | 0.490 | 0.069 |

| | | | | | | |
|---|---|---|---|---|---|---|
| Test A non-correct no-reactions | 0.026 | 0.289 | 0.603 | 0.068 | 0.451 | 0.075 |
| Test A reaction time | 0.026 | 0.243 | 0.001 | 0.509 | 0.014 | 0.246 |
| Test B correct no-reactions | 0.560 | 0.062 | 0.288 | 0.141 | 0.738 | 0.034 |
| Test B non-correct no-reactions | 0.367 | 0.106 | 0.232 | 0.276 | 0.237 | -0.124 |
| Test B reaction time | 0.000 | 0.464 | 0.003 | 0.460 | 0.037 | 0.213 |
